# Influence of bisphenol A and its analog bisphenol S on cocaine- and amphetamine-regulated transcript peptide–positive enteric neurons in the mouse gastrointestinal tract

**DOI:** 10.3389/fnmol.2023.1234841

**Published:** 2023-08-22

**Authors:** Krystyna Makowska, Kainã R. C. Fagundes, Sławomir Gonkowski

**Affiliations:** ^1^Department of Clinical Diagnostics, Faculty of Veterinary Medicine, University of Warmia and Mazury in Olsztyn, Olsztyn, Poland; ^2^Laboratório de Morfofisiologia Animal, Instituto de Biociências, Universidade Estadual Paulista, São Paulo, Brazil; ^3^Department of Clinical Physiology, Faculty of Veterinary Medicine, University of Warmia and Mazury in Olsztyn, Olsztyn, Poland

**Keywords:** endocrine disruptors, bisphenol A, bisphenol S, enteric neurons, digestive tract, mouse

## Abstract

**Introduction:**

Bisphenol A (BPA) is used in large quantities for the production of plastics and is present in various everyday objects. It penetrates living organisms and shows multidirectional adverse influence on many internal organs. For this reason, BPA is often replaced in plastic production by other substances. One of them is bisphenol S (BPS), whose effects on the enteric nervous system (ENS) have not been explained.

**Methods:**

Therefore, the present study compares the influence of BPA and BPS on the number of enteric neurons immunoreactive to cocaine-and amphetamine-regulated transcript (CART) peptide located in the ENS of the stomach, jejunum and colon with the use of double immunofluorescence method.

**Results:**

The obtained results have shown that both bisphenols studied induced an increase in the number of CART-positive enteric neurons, and the severity of changes depended on the type of enteric ganglion, the dose of bisphenols and the segment of the digestive tract. The most visible changes were noted in the myenteric ganglia in the colon. Moreover, in the colon, the changes submitted by BPS are more noticeable than those observed after BPA administration. In the stomach and jejunum, bisphenol-induced changes were less visible, and changes caused by BPS were similar or less pronounced than those noted under the impact of BPA, depending on the segment of the gastrointestinal tract and ganglion type studied.

**Discussion:**

The results show that BPS affects the enteric neurons containing CART in a similar way to BPA, and the BPS impact is even stronger in the colon. Therefore, BPS is not neutral for the gastrointestinal tract and ENS.

## Introduction

Cocaine- and amphetamine-regulated transcript (CART) peptide is an active neuronal substance which was described for the first time in the hypothalamus of a sheep in the early 1980s (Spiess et al., 1981). It was found that the amount of mRNA for the synthesis of this peptide increased after the administration of cocaine and amphetamine, hence the name CART (Douglass et al., 1995).

Since its discovery, CART has been described in many parts of the living organism, including neuronal and endocrine tissues (Douglass et al., 1995; Koylu et al., 1997; Wierup et al., 2007). As regards the nervous system, CART primarily occurs in the brain, where it is an important factor taking part in food intake and the regulation of energy homeostasis (Lau and Herzog, 2014). However, CART has also been found in the peripheral nervous system, including, among others, sensory neurons and nerve fibers innervating endocrine glands in pancreas (Dun et al., 2000; Wierup et al., 2007). The part of the peripheral nervous system where CART is present in significant amounts is the enteric nervous system (ENS).

The ENS is composed of millions of neurons sited in the wall of the gastrointestinal tract from the esophagus to the anus and is characterized by a significant degree of autonomy from the central nervous system (CNS) (Furness, 2000). The enteric neurons are grouped in ganglia, whose number depends on the mammal species and segment of the digestive tract (Furness, 2000). In rodents, two types of enteric ganglia have been found: myenteric ganglion (MG) within the muscular layer between circular and longitudinal muscular fibers and submucous ganglion (SG) in the submucosal layer near the mucosa (Furness, 2012). The enteric neurons are characterized by a significant neurochemical diversity and can produce dozens of active substances that may act as neuromodulators, transporters and/or enzymes. As mentioned above, one of these substances is CART, which has been described in the ENS in various mammal species, including humans (Ekblad, 2006). Interestingly, although the mouse is a very common laboratory animal, knowledge of CART in the ENS of this species is relatively scarce. However, it is known that CART is present in the mouse digestive tract and plays an important role (Ekblad, 2006; Palus et al., 2019). Despite this, the population size and distribution of enteric neurons containing this substance have not yet been described in this species.

It is important to highlight that the precise functions of CART in the ENS are still not fully understood. However, the presence of this peptide within different neurochemical classes of enteric neurons strongly indicates its involvement in multiple functions within the digestive tract (Moffett et al., 2006). It is known that CART is involved in the regulation of gastrointestinal motility and secretory activity (Okumura et al., 2000; Smedh and Moran, 2003; Tebbe et al., 2004) and takes part in feeding modulation and glucose metabolism (Muller et al., 2020). Previous studies describing changes in the population size of CART-positive enteric neurons under the impact of various pathological and toxic factors imply that CART in the ENS is also involved in the neuroprotective and/or regenerative processes (Wu et al., 2006; Bharne et al., 2013; Luo et al., 2013). It is likely that such functions of CART have also been described in the brain, where this substance is recognized as one of the protective factors during ischemic injury (Ahmadian-Moghadam et al., 2018).

One of the toxic factors that is known to influence CART-positive enteric neurons is bisphenol A (BPA) (Szymanska et al., 2018; Makowska and Gonkowski, 2019, 2020; Szymanska and Gonkowski, 2019). BPA is an organic synthetic compound used in various plastics industry branches for the production of epoxy resins and polycarbonates. It is present in various items used in everyday life, such as bottles, furniture, electronic equipment, clothes and even dental fillings (Vandenberg et al., 2007; Staples et al., 2010; Mikolajewska et al., 2015). BPA can penetrate from plastic to the various elements of the environment (such as: air, soil, water) and food. The widespread use of BPA in industry and high environmental pollution by this substance pose a serious threat to the health of humans and animals. It has been shown that BPA enters the organisms mainly with food through the digestive tract but also through the respiratory system and skin (Vandenberg et al., 2007). Due to its structural similarity to estrogen, BPA binds to estrogen receptors throughout the body, shows an endocrine disputing activity and causes functional disturbances in the nervous, reproductive, endocrine and immune systems (Vandenberg et al., 2007; Rubin, 2011; Mikolajewska et al., 2015). The substance also causes serious changes in the intestines, including modification of the permeability of the mucosa, inflammatory changes and disturbances in motility. Exposure to BPA may also result in a higher risk of neoplasm, hypertension and diabetes (Han and Hong, 2016; Chludzińska et al., 2018; Farrugia et al., 2021; Khan et al., 2021).

The strong endocrine-disrupting properties of BPA have led many countries to restrict its use, especially in the production of items intended for infants and children and materials that come into contact with food and drinking water (Mikolajewska et al., 2015). In the production of such items, BPA is replaced by other compounds with similar properties, including bisphenol S (BPS) – one of the analogs of BPA in common use (Bousoumah et al., 2021). Until recently, BPS has been regarded as being completely harmless for living organisms and was widely used for the production of “BPA-free” plastics used in baby pacifiers, bottles and toys (Žalmanová et al., 2016). However, more recent studies indicate that BPS also shows endocrine-disrupting properties and affects living organisms similarly to BPA. Exposure to BPS results in functional disorders and morphological changes in many internal organs, including the reproductive, endocrine, and immune systems (Ji et al., 2013; Žalmanová et al., 2016; Dong et al., 2018; Qiu et al., 2018a,b). In the light of previous studies it is known that BPS among others promotes endometrial epithelial cell proliferation and migration in the uterus and increases absolute and relative wet weights of this organ (Siracusa et al., 2018; Benjamin et al., 2023), changes the hormonal levels in the follicular and oviduct fluids (Téteau et al., 2022), shows proinflammatory and immunomodulatory effects by up-regulation the production of free radicals and cytokine expression (Qiu et al., 2018a,b) and impairs testosterone synthesis in testicular tissues (Wang et al., 2023) Some studies have also reported that BPS may lead to hypertension, obesity, diabetes and even cancer (Apau et al., 2018; Huang et al., 2019; Rancière et al., 2019; Xu et al., 2019; Zhang et al., 2023). Moreover, some observations strongly suggest that the endocrine-disrupting impact of BPS in some organs may be stronger than the effects observed under BPA influence (Pang et al., 2019; Wang C. et al., 2021). It is also known that BPS affects the gastrointestinal tract, indicating oxidative damage and inflammatory effects (Wang C. et al., 2021), affecting the gut microbiome (Wang Y. et al., 2021), impairing immune functions at intestinal level (Malaisé et al., 2020) and reducing the expression of genes responsible for glucose metabolism (Rezg et al., 2019). BPS also affects the ENS changing expression of some neuronal factors, but knowledge of these items is rather limited (Makowska and Gonkowski, 2023).

Considering the above, the aim of the present study was to describe for the first time the exact localization of neurons containing CART in the particular types of enteric ganglia in various segments of the mouse gastrointestinal tract. However, this study also aimed to compare the influence of BPA and its analog BPS on the population of CART–positive enteric neurons. Such a comparison has never been made before, and the present study may contribute to a better understanding of the harmful influence of BPS on the gastrointestinal tract and the functions of CART in the ENS under the impact of toxic factors.

## Materials and methods

The present experiment was carried out using 35 CD1 strain mice. They were adult animals (aged 3 months at the start of the experiment) of both genders with a weight of about 30 g. During the experiment, the animals were kept in standard laboratory conditions at the animal house of the Faculty of Veterinary Medicine (University of Warmia and Mazury in Olsztyn, Poland). The conditions of keeping the animals were as follows: temperature: 22 ± 2.0°C, humidity: 55 ± 10%, light–dark cycle: 12:12 h, food and water: *ad libitum*. All experimental activities the mice were subjected to during the study were approved by the Local Ethical Committee on Experimental Animals in Olsztyn - Poland (Decision No. 46/2019).

The mice were randomly divided into five groups of seven animals each: a control group (C group) which both bisphenols were not administered; the BPA 1 group, in which BPA at a dose of 5 mg/kg body weight (b.w.)/day was given; the BPA2 group, treated with BPA a dose of 50 mg/kg b.w./day; the BPS 1 group, in which animals were subjected to BPS at a dose of 5 mg/kg b.w. and the BPS2 group, in which BPS at a dose of 50 mg/kg b.w./day was administered. Bisphenols were administered in drinking water according to the methods described in previous studies by Dobrzynska et al. (2018) and Rezg et al. (2018), and the dosage was based on the assumption that the mice drink about 2 mL/10 g b.w. per day (Chen et al., 2018). Based on previous studies, the lower dose of bisphenols used in this study is considered the no-observed-adverse-effect-level (NOAEL) for BPA, and the higher dose is the lowest observed adverse effect level (LOEL) for BPA in the mice (Tyl et al., 2002; Choi et al., 2010; Zielinska et al., 2018). BPA and BPS were given in the same doses to compare the effects of both compounds.

Bisphenols were administered for 3 months. Thereafter, all mice were euthanized by decapitation. Immediately after death, fragments of the stomach (the part of the gastric corpus), jejunum (from the place located about 15 cm behind the stomach) and colon (located about 10 cm up to the anus) were collected and fixed overnight in 4% buffered paraformaldehyde (pH 7.4) at 4°C. The tissues were then rinsed in 0.1 M phosphate buffer (pH 7.4) for 3 days at 4°C with a daily buffer exchange. After this period, the tissues were stored in an 18% phosphate-buffered sucrose solution at 4°C for at least 3 weeks. The fragments of the digestive tract were then frozen at –20°C, and sections were cut using the cryostat (Microm, HM 525, Walldorf, Germany) with a thickness of 10 μm and placed on microscopic slides. In this form, the tissues were stored at –20°C for further studies.

Sections of the digestive tract were subjected to standard double immunofluorescent technique according to the method described in previous studies (Makowska and Gonkowski, 2023). In brief, this method was as follows. Slides with tissue sections after removal from the freezer were dried for 1 h (at room temperature rt). They were then incubated with “blocking solution” (10% normal goat serum, 0.1% bovine serum albumin, 0.01% NaN3, 0.25% Triton X-100 and 0.05% thimerosal in PBS) for 1 h to prevent non-specific labeling. Subsequently, tissues were incubated overnight in a humidity chamber with a mixture of two primary antibodies directed against pan-neuronal marker protein gene product 9.5 – PGP 9.5 (mouse antibody from Biogenesis Ltd., Poole, UK, catalog no. 7863–2004, working dilution 1:1000) and CART peptide (rabbit antibody from Phoenix, Aachen, Germany, catalog no. H-003-61, working dilution 1:8000). The following day, the tissues were incubated for 1 h with a mixture of species-specific secondary antibodies conjugated with fluorochromes to visualize “antigen – primary antibody” complexes. The following secondary antibodies were used in the study: donkey anti-mouse IgG conjugated with Alexa fluor 488 and donkey anti-rabbit IgG conjugated with Alexa fluor 546 (both from Invitrogen, Carlsbad, CA, USA, both in a working dilution of 1:1000). The tissue sections were then treated with buffered glycerol and covered with coverslips. Between each step of labeling, tissue sections were rinsed in PBS for 30 min with a PBS change every 10 min. In order to exclude non-specific staining, typical tests were used, i.e., pre-absorption of antibodies with appropriate antigens, as well as omission and replacement of primary antibodies by non–immune sera.

Labeled tissue fragments were analyzed under a BX51 microscope equipped with appropriate filters (Olympus, Tokyo, Japan). The proportion of neuronal cells expressing CART was determined by assessing a minimum of 300 cells that tested positive for the neuronal marker PGP 9.5. These cells were then evaluated for the presence of CART, and their numbers were converted into percentages. In this calculation, the total count of PGP-9.5-positive cells was considered 100%. For the purpose of avoiding double evaluation of the same neurons, sections of the gastrointestinal tract evaluated under the microscope were located at least 200 μm from each other.

To evaluate the influence of bisphenols on the total number of the enteric neurons immunoreactive to PGP 9.5, as well as neurons containing CART, number cells immunoreactive to PGP 9.5 and CART in the myenteric and submucous ganglia in each animal was evaluated. Cells were counted in 50 ganglia (of each type) located on at least 10 slides (sections of the gastrointestinal tract were located at least 200 μm apart).

The obtained results were depicted as mean ± SEM. Statistical analysis was performed with an ANOVA test (Statistica 13, StatSoft, Inc., Cracow, Poland) with statistical significance at *p* ≤ 0.05.

## Results

Cocaine- and amphetamine-regulated transcript-positive neuronal cells were noted in all segments of the gastrointestinal tract both in physiological terms and after the administration of bisphenols (Table 1).

**Table 1:** The distribution of enteric neurons immunoreactive to cocaine- and amphetamine-related transcript (CART) peptide in the porcine stomach, jejunum, and colon.

Stomach						
		C	BPA1	BPA2	BPS1	BPS2
MG	A	3523/1367	3536/1198	3529/1287	3503/960	3550/1160
	B	25.42 ± 0.96%*	33.88 ± 1.31%*	36.47 ± 0.44%*	27.34 ± 1.41%	32.68 ± 0.27%*
SG	A	3482/557	3532/932	3523/1087	3526/866	3519/863
	B	20.80 ± 0.98%*	26.38 ± 1.12%*	30.85 ± 0.69%*	24.56 ± 1.13%*	24.52 ± 1.01%*

Jejunum						
		C	BPA1	BPA2	BPS1	BPS2
MG	A	3532/707	3525/960	3527/1084	3529/833	3541/921
	B	20.00 ± 1.13%*	27.24 ± 0.67%*	30.76 ± 1.97%*	23.59 ± 0.78%*	26.00 ± 1.16%*
SG	A	3482/634	3515/812	3525/964	3494/838	3526/876
	B	18.19 ± 0.92%*	23.10 ± 0.55%*	27.35 ± 0.32%*	23.96 ± 0.65%*	24.84 ± 0.87%*

Colon						
		C	BPA1	BPA2	BPS1	PS2
MG	A	3523/1367	3528/2168	3524/2249	3522/2223	3513/2383
	B	38.79 ± 1.62%*	61.43 ± 2.23%*	63.81 ± 2.05%*	63.12 ± 1.23%*	67.82 ± 1.75%*
SG	A	3479/557	3523/803	3515/1060	3520/1346	3491/1354
	B	16.01 ± 0.95%*	22.79 ± 1.49%*	30.15 ± 2.34%*	38.23 ± 2.06%*	38.78 ± 1.94%*

The presented data are a mean value of 7 animals from each group. A, the number of cells PGP+/CART+ included into the study. B, the percentage of CART+ neurons (mean ± SEM), where the number of PGP+ neurons was considered as 100%. MG, myenteric plexus. SG, submucous plexus. Statistically significant differences (*p* < 0.05) between control animals (C group) and animals receiving low dose (BPA1 group) or high dose (BPA2 group) of bisphenol A as well as between C group and animals treated with low dose (BPS1 group) or high dose (BPS2 group) of bisphenol S are marked with *.

In control animals, the percentage of neurons containing CART in the MG varied from 20.00 ± 1.13% of all PGP 9.5 – positive cells in the jejunum to 38.79 ± 1.62% in the colon. In the SG of the control animals, the percentage of CART-positive cells was lower compared to the number of such cells in the MP of the same segment and amounted to 16.01 ± 0.95% in the colon to 20.80 ± 0.98% in the stomach.

**Figure 1:**
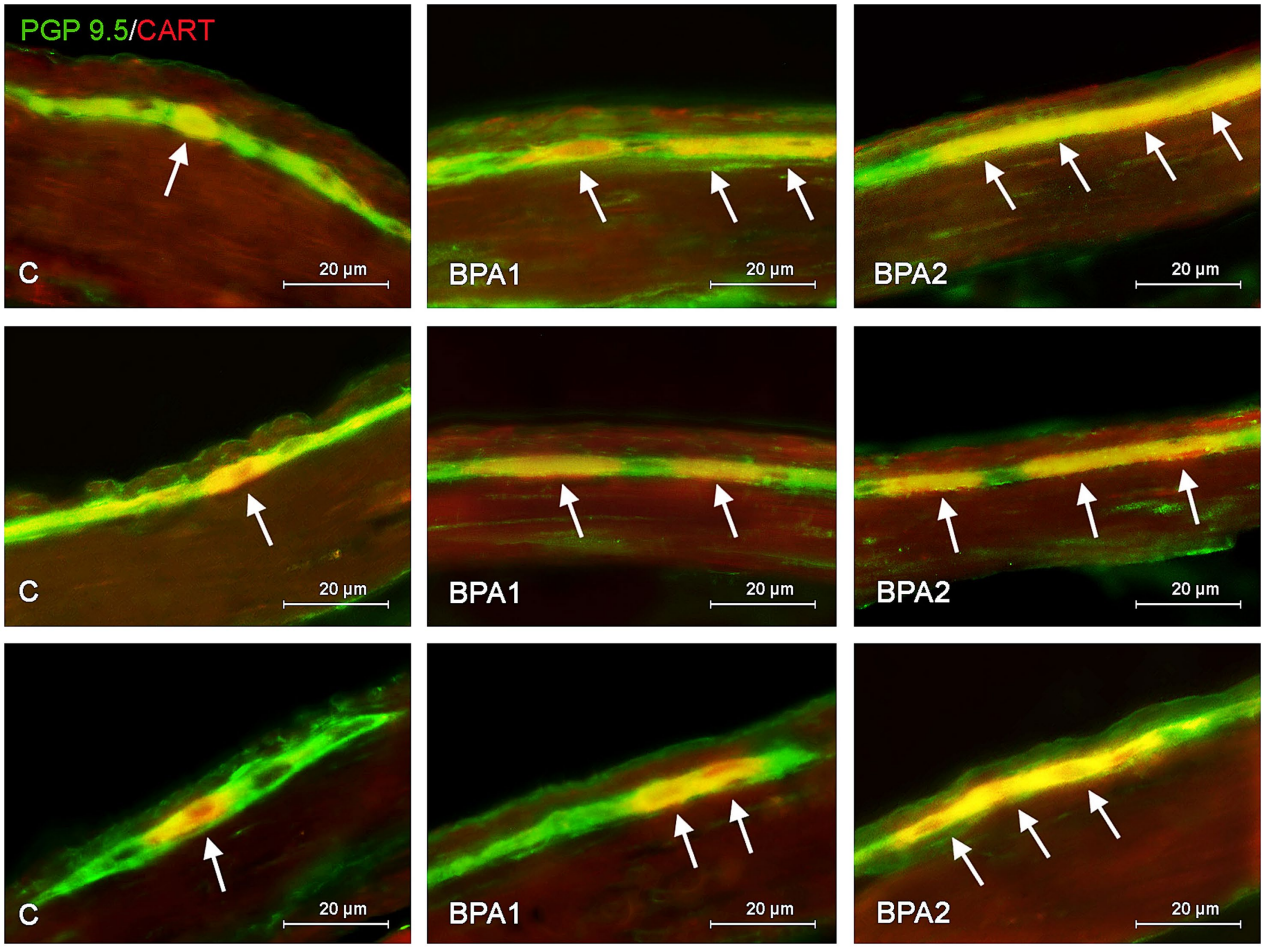
Distribution pattern of neuronal cells immunoreactive to protein gene-product 9.5 (PGP 9.5) – used as pan neuronal marker and cocaine and amphetamine regulated transcript (CART) peptide in the myenteric plexus of porcine stomach **(first row)**, jejunum **(second row)**, and colon **(third row)** under physiological conditions (C) and after administration of small dose (BPA1) and high dose (BPA2) of bisphenol A. The pictures are the result of the overlap of both staining. The arrows are pointing neurons immunoreactive for both – PGP 9.5 and CART.

**Figure 2:**
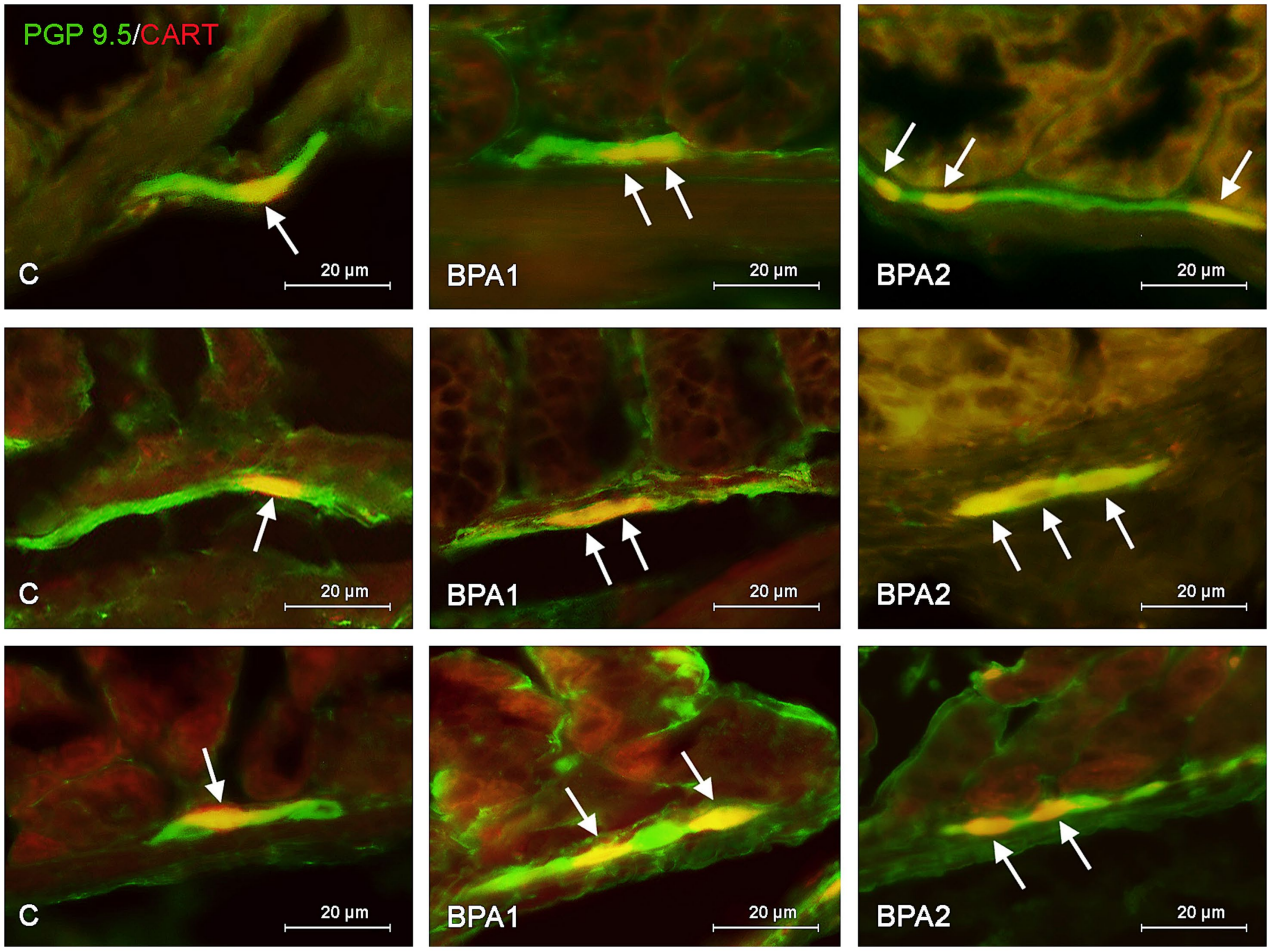
Distribution pattern of neuronal cells immunoreactive to protein gene-product 9.5 (PGP 9.5) – used as pan neuronal marker and cocaine and amphetamine regulated transcript (CART) peptide in the submucous plexus of porcine stomach **(first row)**, jejunum **(second row)**, and colon **(third row)** under physiological conditions (C) and after administration of small dose (BPA1) and high dose (BPA2) of bisphenol A. The pictures are the result of the overlap of both staining. The arrows are pointing neurons immunoreactive for both – PGP 9.5 and CART.

Administration of BPA caused an increase in the percentage of CART-positive cells in both types of enteric ganglia studied (Figures 1, 2). In the MG (Figure 1), the most visible changes were found in the colon, in which the number of cells immunoreactive to CART increased from 38.79 ± 1.62% of all PGP 9.5-positive cells in the control group to 61.43 ± 2.23% and 63.81 ± 2.05% under the influence of lower and higher dose of BPA, respectively. Changes noted under the influence of BPA in the MG of other segments of the gastrointestinal tract were less visible. The percentage of CART-positive cells in the MG of the stomach increased from 25.42 ± 0.96% in the control animals to 33.88 ± 1.31% under a lower dose of BPA and to 36.47 ± 0.44% under a higher dose of BPA. In turn, the percentage of cells immunoreactive to CART in the jejunal MG achieved 27.24 ± 0.67% and 30.76 ± 1.97% in BPA1 and BPA2 groups, respectively.

In the SG (Figure 2), both doses of BPA caused an increase in the percentage of CART–positive neurons. Intensification of changes under the impact of lower doses of BPA was similar in all segments of the gastrointestinal tract studied. The percentage of neurons containing CART increased from 20.80 ± 0.98% to 26.38 ± 1.12% in the stomach, from 18.19 ± 0.92% to 23.10 ± 0.55% in the jejunum and from 16.01 ± 0.95% to 22.79 ± 1.49% in the colon. In turn, the higher dose of BPA causes the most visible changes in the SG of the colon, where the percentage of CART–positive cells achieved 30.15 ± 2.34% of all PGP 9.5-positive cells (the increase from 16.01 ± 0.95% in the control animals). Less-visible changes were noted in the SG of the stomach (increase to 30.85 ± 0.69%) and in the jejunum (increase to 27.35 ± 0.32%).

**Figure 3:**
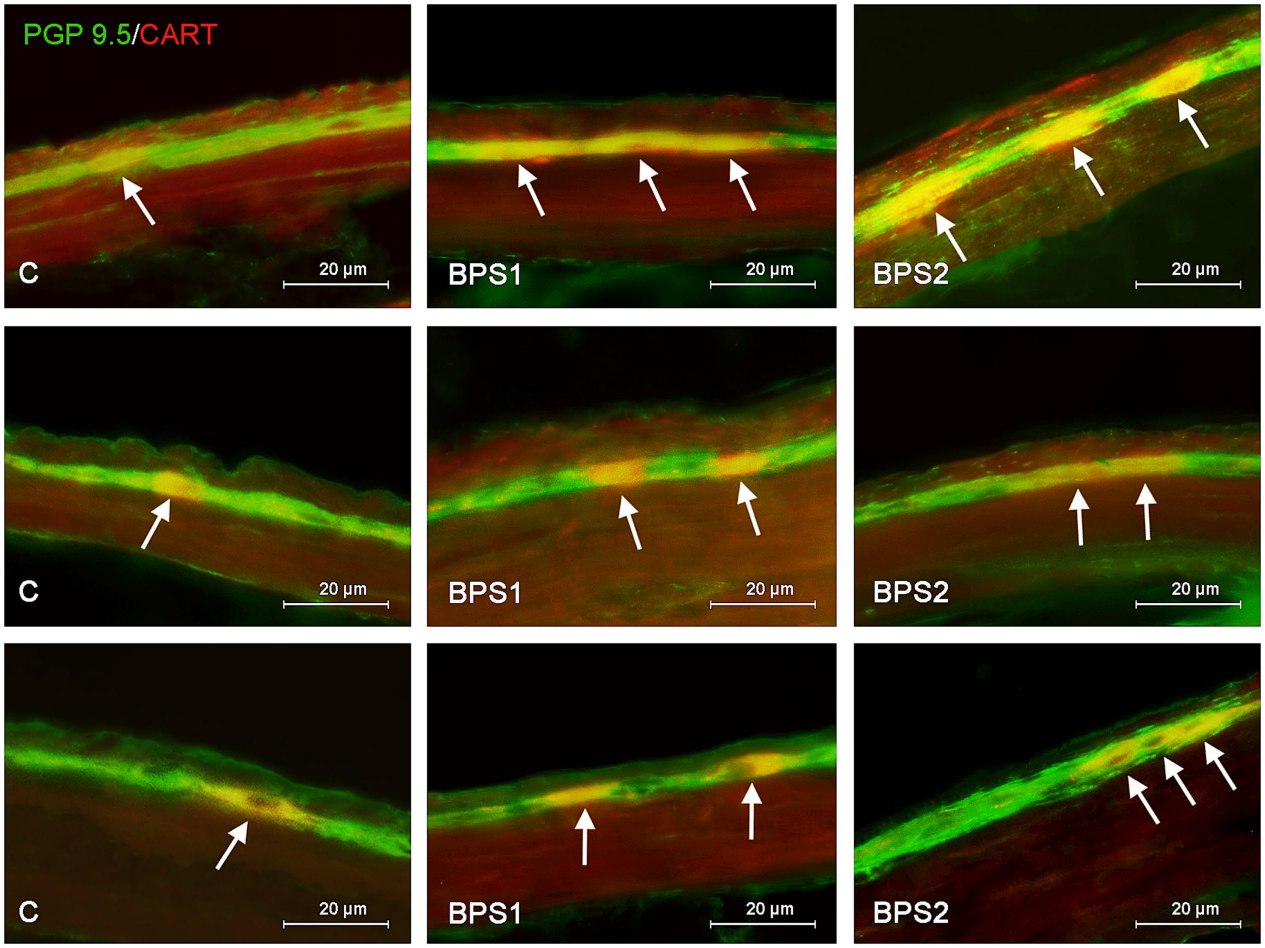
Distribution pattern of neuronal cells immunoreactive to protein gene-product 9.5 (PGP 9.5) – used as pan neuronal marker and cocaine and amphetamine regulated transcript (CART) peptide in the myenteric plexus of porcine stomach **(first row)**, jejunum **(second row)**, and colon **(third row)** under physiological conditions (C) and after administration of small dose (BPS1) and high dose (BPS2) of bisphenol S. The pictures are the result of the overlap of both staining. The arrows are pointing neurons immunoreactive for both – PGP 9.5 and CART.

**Figure 4:**
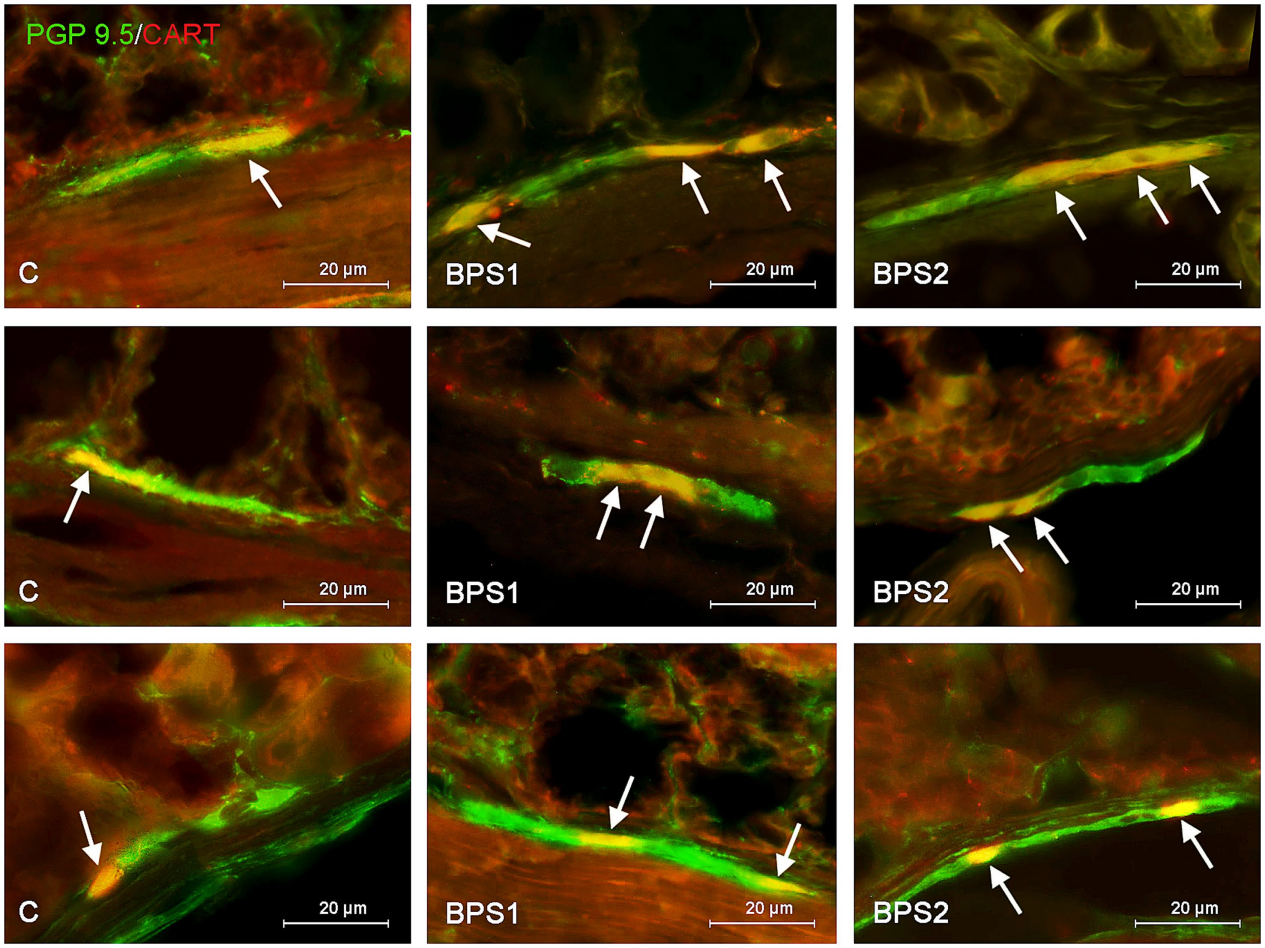
Distribution pattern of neuronal cells immunoreactive to protein gene-product 9.5 (PGP 9.5) – used as pan neuronal marker and cocaine and amphetamine regulated transcript (CART) peptide in the submucous plexus of porcine stomach **(first row)**, jejunum **(second row)**, and colon **(third row)** under physiological conditions (C) and after administration of small dose (BPS1) and high dose (BPS2) of bisphenol S. The pictures are the result of the overlap of both staining. The arrows are pointing neurons immunoreactive for both – PGP 9.5 and CART.

Administration of BPS (similar to BPA) caused an increase in the percentage of neurons containing CART (Figures 3, 4). In the colon, changes noted under the impact of BPS were more visible than those observed after the treatment with BPS. In the MG, administration of BPS caused an increase in the percentage of CART–like immunoreactive cells from 38.79 ± 1.62% in the control animals to 63.12 ± 1.23% under the impact of a lower dose of BPS and to 67.82 ± 1.75% after administration of higher doses of BPS. In the colonic SG, the percentage of cells containing CART increased from 16.01 ± 0.95% in the control mice to 38.23 ± 2.06% and 38.78 ± 1.94% in BPS 1 and BPS 2 groups, respectively.

In the stomach and jejunum, the changes caused by BPS were similar or less visible than those noted under the exposure of BPA, depending on the segment of the gastrointestinal tract and ganglion type studied. In the gastric MG, the lowest dose of BPS did not cause statistically significant changes in the percentage of CART–positive cells, and the administration of a higher dose resulted in the increase in the percentage of those cells to 32.68 ± 0.27% of all cells containing PGP 9.5. On the other hand, in the SG of the stomach, BPS caused an increase (less visible than that observed in the case of BPA) in the percentage of CART–positive neurons from 20.80 ± 0.98% in the control mice to 24.56 ± 1.13% after the administration of a lower dose and to 24.52 ± 1.01% under the impact of a higher dose.

In the jejunal MG, the impact of BPS was also less visible than that seen after the administration of BPA. The treatment with this compound resulted in an increase in the percentage of CART–like immunoreactive neurons from 20.00 ± 1.13% to 23.59 ± 0.78% and 26.00 ± 1.16% in BPS 1 and BPS2 groups, respectively. In turn, in the SG of the jejunum a lower dose of BPS caused an increase in the percentage of CART-like immunoreactive cells from 18.19 ± 0.92% to 23.96 ± 0.65%, and this change was similar to that noted under the impact of a lower dose of BPA. In animals exposed to a higher dose of BPS, the percentage of neurons containing CART in the jejunal SG achieved 24.84 ± 0.87%, which indicated that a higher dose of BPS affected the population of CART–positive cells to a lesser extent than a higher dose of BPA.

Moreover, during the present investigation noticed that both bisphenols affect the entire population of enteric neurons counted in 50 enteric ganglia. Generally, both low and high doses of BPA and BPS caused a statistically significant decrease in the number of neuronal cells in both studied plexuses but the level of those changes depended on the dose, type of bisphenol, type of studied plexus and the part of the GI tract (Table 2).

**Table 2:** Mean number of enteric neurons (±SEM) counted in 50 enteric ganglia in control animals (C), after administration of BPA in low (BPA 1) and high dose (BPA2) and after administration of BPS in low (BPS1) and high (BPS2) dose.

Stomach					
	C	BPA1	BPA2	BPS1	BPS2
MG	839.71 ± 14.35	790.29 ± 8.39*	680.29 ± 17.64*	802.71 ± 6.34*	743.57 ± 8.93*
SG	282.86 ± 6.46	250.14 ± 4.97*	224.29 ± 4.64*	258.14 ± 3.53*	226.71 ± 5.28*

Jejunum					
	C	BPA1	BPA2	BPS1	BPS2
MG	1103.29 ± 5.87	1041.86 ± 8.30*	977.14 ± 17.62*	1058.57 ± 6.52*	1010.57 ± 5.18*
SG	292.43 ± 3.32	268.43 ± 4.09*	233.14 ± 4.51*	275.14 ± 3.52*	239.71 ± 3.19*

Colon					
	C	BPA1	BPA2	BPS1	BPS2
MG	1266.71 ± 5.21	1190.57 ± 4.82*	1093.86 ± 8.39*	1000.71 ± 2.91*	985.43 ± 4.16*
SG	262.29 ± 3.02	220.86 ± 2.26*	202.43 ± 1.99*	226.86 ± 3.36*	223.57 ± 4.25*

Statistically significant differences between particular groups of animals and control group are marked with *.

In the present study the effect of bisphenols on the total population of CART+ neurons was also evaluated (Table 3). Under the influence of both low and high doses of BPA and BPS noticed an statistically significant increase in the number of neuronal cells immunoreactive to CART in every part of the ENS studied. The observed changes were more visible in case of higher dose of both studied bisphenols.

**Table 3:** Mean number of enteric neurons immunoreactive to cocaine- and amphetamine-regulated transcript (CART) peptide (±SEM) counted in 50 enteric ganglia in control animals (C), after administration of BPA in low (BPA 1) and high dose (BPA2) and after administration of BPS in low (BPS1) and high (BPS2) dose.

Stomach					
	**C**	**BPA1**	**BPA2**	**BPS1**	**BPS2**
MG	215.00 ± 2.20	238.71 ± 4.05*	253.00 ± 3.24*	223.29 ± 2.76*	236.86 ± 2.56*
SG	53.71 ± 1.15	89.71 ± 1.67*	68.86 ± 1.47*	64.14 ± 0.91*	57.71 ± 0.97*

Jejunum					
	C	BPA1	BPA2	BPS1	BPS2
MG	232.86 ± 4.93	273.14 ± 4.6*	299.43 ± 5.33*	256.57 ± 2.55*	262.57 ± 1.56*
SG	53.29 ± 0.87	58.86 ± 1.77*	62.43 ± 1.46*	65.14 ± 0.94*	59.57 ± 0.92*

Colon					
	C	BPA1	BPA2	BPS1	BPS2
MG	492.29 ± 2.41	672.43 ± 12.91*	686.86 ± 4.31*	629.71 ± 1.82*	659.86 ± 2.76*
SG	42.71 ± 1.11	48.00 ± 0.53*	60.00 ± 0.69*	83.43 ± 2.22*	83.29 ± 2.11*

Statistically significant differences between particular groups of animals and control group are marked with *.

## Discussion

This is the first report concerning the exact distribution of CART–positive cells in the ENS of the mouse stomach and intestine. During the present study, CART–like immunoreactive neurons were found in all segments of the digestive tract studied, which is in agreement with previous observations conducted on other mammal species, where this peptide was found in different segments of the gastrointestinal tract from the esophagus to the rectum in all types of enteric ganglia (Ekblad, 2006). This fact, allied with the present results, strongly suggests the importance and manifold roles of this peptide in the regulation of the gastrointestinal tract. The diverse roles of CART in the ENS are supported by the fact that previous publications described the occurrence of this peptide in various classes of enteric neurons, producing a wide range of other neurotransmitters (Couceyro et al., 1998; Wojtkiewicz et al., 2012; Bulc et al., 2014; Rękawek et al., 2015).

However, it should be pointed out that the roles of CART in the ENS are not fully explained. Previous investigations have shown that CART may participate in the regulation of gastrointestinal secretory activity (Okumura et al., 2000; Smedh and Moran, 2003; Tebbe et al., 2004) and influence intestinal motility. This is confirmed by the presence of CART–positive neurons both in the MG – mainly responsible for intestinal motor activity and in the SG – primarily regulating the intestinal secretion observed in the present study and previous investigation (Ekblad, 2006; Rękawek et al., 2015; Makowska and Gonkowski, 2020). Moreover, it is known that CART occurs in viscerofugal enteric neurons polysynaptically associated with the pancreas and liver through prevertebral ganglia and participates in the regulation of food intake and glucose metabolism, affecting blood glucose levels (Muller et al., 2020). In contrast, differences in the number of CART–like immunoreactive neurons among particular regions studied were observed in both the present experiment and previous investigations (Ekblad, 2006; Szymanska et al., 2018; Szymanska and Gonkowski, 2018; Makowska and Gonkowski, 2020) which strongly suggests that the exact functions of this peptide depend on the segment of the digestive tract.

Previous studies have also described the changes in the population size of CART–positive enteric neurons under the impact of various pathological factors, including inflammation, nerve fiber damage, experimental diabetes, neoplastic processes, hypertension and various toxic substances (Apau et al., 2018; Huang et al., 2019; Rancière et al., 2019; Xu et al., 2019; Zhang et al., 2023). These observations, in which pathological factors generally caused an increase in the number of CART-like immunoreactive neurons in the ENS, strongly suggest that this peptide takes part in adaptive and/or protective reactions in the gastrointestinal tract, leading to homeostasis maintenance in conditions changed by the acting stimulus. Observations concerning the possible neuroprotective roles of CART have been performed during *in vitro* studies on the myenteric neuron cultures (Ekblad, 2006). In a study by Lin et al., the increase in the synthesis of CART by culturing myenteric neurons and the influence of CART on the synthesis of VIP – a well-known neurotrophic factor – was observed (Lin et al., 2003). However, the addition of CART to neuronal cultures had little effect on neuronal survival (Ekblad, 2006). Nevertheless, it is more plausible that CART plays a role in neuroprotective responses within enteric neurons rather than exerting neuroprotective effects observed in other regions of the nervous system, particularly the central nervous system (Vrang, 2006). However, the mechanisms of these processes in the ENS are still unknown, and fluctuations in the number of CART-positive neuronal cells may be connected with changes in CART production on transcriptional, translational or metabolic levels, as well as disturbances in the intracellular transport of this peptide.

One of the chemical factors which may affect the number of CART-positive enteric neurons is BPA. Previous studies have shown that this compound, even in relatively low doses, causes an increase in the number of such neurons in the porcine gastrointestinal tract (Szymanska and Gonkowski, 2018; Makowska and Gonkowski, 2020), which is also confirmed by the present results. Moreover, the present study clearly shows that a BPS – BPA analog, until recently considered neutral for living organisms, also affects CART-containing neurons in the mouse ENS. This is in agreement with more recent studies, which have found that BPS shows endocrine-disrupting properties similar to BPA (Ji et al., 2013; Žalmanová et al., 2016; Dong et al., 2018; Qiu et al., 2018a,b) and, in some cases, BPS impact is even stronger than BPA (Pang et al., 2019; Wang C. et al., 2021). Such a situation has been noted in the present study in the colon, in which the administration of BPS caused more visible changes in the number of CART-positive neurons.

Although the BPS in mice did not change the hypothalamic *cart* mRNA levels (Rezg et al., 2018), the current results showed that both BPA and BPS affected enteric neuron CART synthesis and, consequently, its activity. While the ENS primarily communicates with the CNS through the vagus nerve, it is important to note that enteric neurons function autonomously and can exhibit diverse responses and mechanisms owing to their unique characteristics (Furness et al., 1995). These results demonstrate and reinforce how the enteric neurons serve as useful morpho-functional biomarkers for toxicological and ecotoxicological assessments (Marinsek et al., 2018, 2022).

The exact mechanisms by which bisphenols affect CART-positive enteric neurons are not clear. Changes may be connected with the direct neurotoxic impact of these compounds on the neurons, which, in the light of previous studies, can be manifested in different ways, including disturbances in synaptic functions and synaptogenesis, mitochondrial damage, impairment of ion transport and homeostasis as well as disturbances in the development of nerve fibers (Larsen and Hunter, 2006; Mao et al., 2007, 2012; Lau and Herzog, 2014; Lau et al., 2018; Singh et al., 2021). Considering this, the increase in the number of CART-positive neurons noted in the present study may result from the above-discussed neuroprotective functions of CART, which have been noted both in the enteric neurons (Ekblad, 2006) and other parts of the nervous system (Mao et al., 2007; Lau and Herzog, 2014; Jiao et al., 2018).

Therefore, on one side observed changes may result from mentioned above impact of bisphenols on the various stages (transcriptional, translational, or metabolic) of synthesis of CART in neurons, but on the other hand they may be connected with neurotoxic effects of bisphenols on neurons, which do not contain CART. In this case the increase of the percentage of CART – immunoreactive neurons is only seeming and results from reduction in the total number of neurons under neurotoxic influence of BPA and BPS. Results obtained in this investigation, in which the total number of the enteric neurons under the impact of bisphenols seem to confirm this thesis and neurotoxic effect of bisphenols on various types of the enteric neurons. However, on the other hand the increase in the total number of CART-positive neurons was also noted in the present study under the impact of bisphenols. Therefore, it is highly probable that changes noted in the present study result both from the bisphenol – induced decrease in the total number of the enteric neurons and increase in the production of this substance.

However, the neurotoxic properties of bisphenols may not be the only reason for the observed changes. They may also result from the influence of bisphenols on intestinal motility. It is known that these compounds have relaxing effects on the smooth muscles and cause the inhibition of intestinal motor activity (Sarkar et al., 2014, 2016; Gonkowski, 2020). In turn, CART may inhibit nitric oxide–dependent smooth muscle relaxation and, in consequence, it is a factor stimulating intestinal motility (Rivera et al., 2011; Szymanska et al., 2018; Calka, 2019). Therefore, the increase in the number of neurons containing CART may have a compensatory character and be a reaction to the inhibition of intestinal motility under the influence of bisphenols. Another reason for the changes noted in the present study may be connected with the relatively well-known proinflammatory and immunomodulatory properties of bisphenols (Qiu et al., 2018a,b; Rytel et al., 2019; Gonkowski, 2020; Parrado et al., 2023). Although the doses of bisphenols studied in the present investigation were rather low and did not cause visible inflammatory changes, it cannot be excluded that the increase in the number of CART-positive neurons resulted from the first response to subclinical inflammation. It is more likely that CART is a factor which may modulate immune cell activity (Vandenberg et al., 2007; Rubin, 2011; Mikolajewska et al., 2015). It cannot be ruled out that changes in the number of CART–positive enteric neurons are also caused by the endocrine- and metabolic-disrupting properties of bisphenols. Based on previous studies, it is known that bisphenols affect the activity of insulin and other hormones and contribute to metabolic disorders, the effect of which is the disruption of blood glucose homeostasis, leading to obesity and diabetes (Apau et al., 2018; Huang et al., 2019; Rancière et al., 2019; Xu et al., 2019; Zhang et al., 2023). In turn, CART is one of the main factors in the central nervous system which regulates food intake in the organism (Lau and Herzog, 2014; Muller et al., 2020). Moreover, it is known that CART–positive enteric neurons (belonging to the class of viscerofugal neurons) may affect liver and pancreas activity and take part in the regulation of blood glucose levels (Muller et al., 2020). Therefore, it seems very likely that the metabolic disrupting properties of bisphenols may contribute to the changes noted in the present study. In conclusion, the present study, for the first time, compared the influence of BPA and its analog BPS on the number of CART–positive enteric neurons in various segments of the mouse digestive tract. The results clearly indicate that both bisphenols increase the number of enteric neuronal cells containing CART, and the severity of changes depends on the type of enteric ganglia, the segment of the gastrointestinal tract and the dose of bisphenols. In the colon, changes observed under the influence of BPA are even more visible than those observed after the administration of BPA, which clearly demonstrates that BPS is not neutral for the enteric neurons. The exact mechanisms of the observed changes are difficult to explain due to the multidirectional harmful effects of bisphenols and the unclear functions of CART in the ENS. The changes observed in the current study may result from the neurotoxic and/or metabolic disrupting properties of bisphenols, but they may also be connected with the influence of these compounds on intestinal motility and immune cells. Therefore, further comprehensive research is needed to clarify all aspects related to the involvement of CART in the gastrointestinal response to bisphenol exposure.

## Data availability statement

The raw data supporting the conclusions of this article will be made available by the authors, without undue reservation.

## Ethics statement

The animal study was approved by Local Ethical Committee on Experimental Animals in Olsztyn - Poland (Decision No. 46/2019). The study was conducted in accordance with the local legislation and institutional requirements.

## Author contributions

KM and SG: conceptualization and data curation. KM: formal analysis, funding acquisition, visualization, and writing – original draft. KM, KF, and SG: investigation. KM and KF: methodology. SG: supervision. KF and SG: writing – review and editing. All authors contributed to the article and approved the submitted version.

## Funding

Publication funded by the National Science Centre in Poland (Grant No. 2018/31/N/NZ7/01252).

## Conflict of interest

The authors declare that the research was conducted in the absence of any commercial or financial relationships that could be construed as a potential conflict of interest.

## Publisher’s note

All claims expressed in this article are solely those of the authors and do not necessarily represent those of their affiliated organizations, or those of the publisher, the editors and the reviewers. Any product that may be evaluated in this article, or claim that may be made by its manufacturer, is not guaranteed or endorsed by the publisher.
